# Digital Fringe Projection-Based Clamping Force Estimation Algorithm for Railway Fasteners

**DOI:** 10.3390/s23063299

**Published:** 2023-03-21

**Authors:** Zhengji Fan, Yingping Hong, Yunfeng Wang, Yanan Niu, Huixin Zhang, Chengqun Chu

**Affiliations:** School of Instrument and Electronics, North University of China, Taiyuan 030051, China

**Keywords:** fastener looseness detection, non-contact methods, point cloud processing, kernel density estimation, rail fastener, railway safety inspection, ridge regression

## Abstract

The inspection of railway fasteners to assess their clamping force can be used to evaluate the looseness of the fasteners and improve railway safety. Although there are various methods for inspecting railway fasteners, there is still a need for non-contact, fast inspection without installing additional devices on fasteners. In this study, a system that uses digital fringe projection technology to measure the 3D topography of the fastener was developed. This system inspects the looseness through a series of algorithms, including point cloud denoising, coarse registration based on fast point feature histograms (FPFH) features, fine registration based on the iterative closest point (ICP) algorithm, specific region selection, kernel density estimation, and ridge regression. Unlike the previous inspection technology, which can only measure the geometric parameters of fasteners to characterize the tightness, this system can directly estimate the tightening torque and the bolt clamping force. Experiments on WJ-8 fasteners showed a root mean square error of 9.272 N·m and 1.94 kN for the tightening torque and clamping force, demonstrating that the system is sufficiently precise to replace manual measurement and can substantially improve inspection efficiency while evaluating railway fastener looseness.

## 1. Introduction

A rail fastener connects the rail to a sleeper and plays an important role in maintaining rail stability and railway safety. However, it may come loose because of environmental interference during installation or the high-frequency impacts and vibration as a long-term effect of high-speed trains, thus affecting the stability of the railway. Common failures of elastic railway clips include spring bar breakage and loose or missing fastening bolts [1]. The railway industry has continued developing each year, as evidenced by increases in the number of routes and higher mileage. Thus, there is an increasing demand for automated batch inspection to assess large numbers of railway fasteners for loosening. Simultaneously, in the overhead system [2] and the railroad bridge [3], measurement of clamping forces is necessary for a variety of infrastructure in railway engineering. At present, such inspection is performed mainly by using (1) vibration signal analysis, (2) image acquisition and recognition analysis, or (3) optical scanning to create a three-dimensional topography and apply feature recognition techniques.

Wei et al. [4] proposed a vibration signal analysis method, using an automatic remote sensing measurement system to detect the state of rail fasteners through wavelet analysis on acceleration signals. However, this method is not suitable for batch inspection of fasteners because it requires prior sensor installation.

For the method of image acquisition and recognition analysis, non-contact detection can be carried out by using this method, to avoid the time and economic cost caused by the installation of many sensors. Gibert et al. [5] proposed an analysis model that combines a histogram of oriented gradient features with a linear support vector machine (SVM) classifier; it can detect whether fasteners are absent or defective, with a detection probability of 98% and a false-alarm rate of 1.23%. Bai et al. [6] proposed an improved classification model based on the faster region-based convolutional neural network (Faster R-CNN) combined with support vector data description (SVDD). This algorithm can detect whether fasteners are complete, broken, or missing. At the same time, it can also check whether the fastener is installed at a deflection angle. Dong et al. [7] customized and improved the R-CNN network, using a lightweight backbone network to replace the complex network and incorporating a mixed loss function to solve the small-sample problem of fastener status inspection. Wang et al. [8] used the improved ResNet deep network, which is based on spatial pyramid pooling (SPP), to inspect fastener status. In their study, in order to overcome the imbalance in the number of samples of different types of fasteners, virtual image technology was used to simulate the defect state of fasteners, thus expanding the data set and increasing the accuracy rate of state recognition by 2.4%. The above visual methods all use machine learning methods to detect fasteners, which can effectively detect obvious missing and broken fasteners, but cannot detect complete fasteners in a loose state. To address this looseness detection problem, Pan et al. [9] proposed a vision-based bolt looseness detection system with the Internet of Things (IoT).The system realized high-precision bolt angle change detection by visual decoding of a special bar code marker, and the detection accuracy of the system could reach $0.1^{\circ }$. However, additional equipment still needed to be installed to carry out the test. Wang et al. [10] designed a method that can obtain bolt images at any position around the bolt and conduct loosening detection. The method includes perspective transformation of the original image, recognition of numbers by convolutional neural network to locate bolts, and detection of bolt rotation angle by Hough transform. However, this method also has some shortcomings. It can only detect the angle change of a single circle, and it also needs to manually engrave the number on the bolt to realize positioning.

In the third type of method, optical scanning is used to create a three-dimensional topography, and then feature recognition is performed. Mao et al. [1] used a commercial structured-light sensor to obtain the point cloud of the fastener and then used a decision tree to classify the fastener’s defects. At the same time, the centerline of the fastener’s spring bar was extracted and used to evaluate the looseness of the fastener. Dai et al. [11] obtained three-dimensional topography of railway fasteners by linear laser scanning and then used the height gradient oriented histogram and two classifiers to detect defective fasteners. Cui et al. [12] proposed a coupler geometric parameter measurement system based on a two-dimensional laser profiler, which can measure various geometric parameters of the coupler in real time and with high precision; the root mean square error of measurement is 0.3 mm. Zhan et al. [13] developed a railway detection system using a laser, which was based on RailNet (an efficient convolutional neural network) to identify defective fastenings. All these methods use line laser scanning to obtain the 3D topography of the fastener. However, although the line laser is highly accurate, the scanning speed is slow. In contrast, binocular vision methods are much faster. Thus, Sun et al. [14] proposed a fast inspection method based on binocular vision, which can detect the loosening of key bolt components in railway systems and evaluate whether bolts are loose by calculating the distance between bolt caps and mounting surfaces. However, the binocular vision method has relatively high requirements on the shooting objects. In this study, Sun et al. proposed that the test method can only be used for bolts with a clean surface, and it is not suitable for the relatively complex shape of the spring bar fastener [14].

In summary, for the loosening detection of railway fasteners, a method is still needed that can not only quickly carry out non-contact scanning and detection, but also avoid the above binocular vision problems without installing additional devices on the fasteners. In this paper, the 3D reconstruction technology of digital fringe projection is proposed to detect the looseness of railway fasteners. This method is a non-contact optical scanning method [15], which can obtain high precision 3D topography quickly by shooting several projection images and processing the algorithm [16]. Compared with the traditional image method, this method is more suitable for the detection of the railway fastener, which is a complex shape that is not easy to distinguish. Moreover, the geometric information obtained by three-dimensional scanning is more comprehensive and not limited to the features of a certain visual angle. The shooting angle requirement is much lower than the image method, which is more robust in the actual detection process. At the same time, the above looseness detection methods are limited to measuring only the geometric parameters and relative positions of bolts and nuts at present [1,5,11,13], while in the actual evaluation of the tightness of railway fasteners, the tightening torque and buckle pressure of bolts are measured [17,18]. In this paper, based on the three-dimensional topography of railway fasteners obtained by scanning, a clamping force estimation algorithm is designed. The algorithm can effectively predict the fastening pressure and tightening torque of railway fasteners and estimate the loosening situation of railway fasteners.

The rest of this paper is organized as follows: Section 2 introduce the overall design structure of the system. Section 3 introduces the 3D reconstruction system constructed in this paper and the principle of railway fastener clamping force estimation algorithm. Section 4 evaluates the 3D reconstruction system and gives the overall experimental process of buckle pressure prediction and related results analysis. The conclusions are drawn in Section 5.

## 2. System Overview

**Figure 1:**
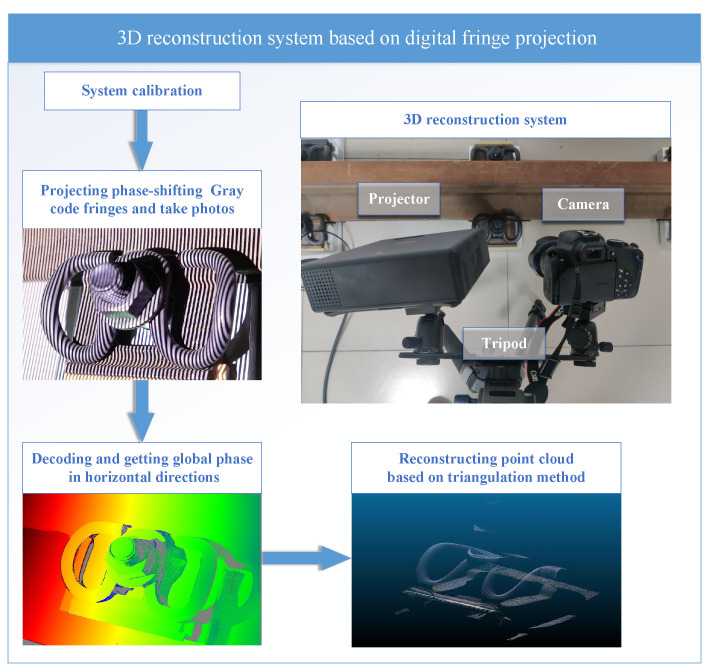
Structured-light reconstruction system.

Based on the 3D measurement technology of digital fringe projection, the railway fastener pressure prediction system is designed as shown in Figure 1. The system includes a projector, a camera, and a gimbal to hold them. In this system, the internal and external parameters of the projector and camera are calibrated in advance, and then the phase shift and gray stripes are projected to the railway couplers through the projector on the left of the figure. Then, the system unwraps the phase after capturing the phase-shift pattern by the camera and obtains the phase order by decoding the Gray code pattern. A reconstruction algorithm based on trigonometry is used to obtain the three-dimensional morphology of railway fasteners from the global phase.

**Figure 2:**
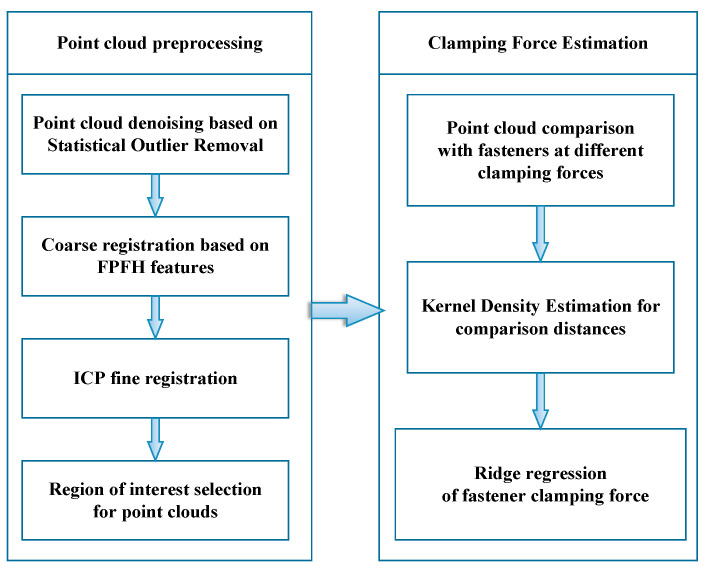
Clamping force estimation algorithm.

Next, the system in Figure 2 preprocesses the point cloud through point cloud denoising, point cloud coarse registration based on fast point feature histograms (FPFH) features of the point cloud, iterative closest point (ICP) fine registration, and the selection of specific areas of the point cloud so that the point cloud always maintains the same position and attitude before comparison and only processes the spring bar part of the fastener. Then, the system makes a horizontal comparison between the model and the point clouds in different clamping states and estimates the probability distribution within a certain interval by kernel density estimation. Finally, through the established ridge regression model, the actual clamping force is estimated.

In recent years, four types of fasteners have generally been used in China’s high-speed rail: WJ-7, WJ-8, W300-1, and SFC [1]. WJ-8 was used as the experimental and test fastener in this study.

## 3. Methodology

### 3.1. Structured-Light Reconstruction System

In the 3D reconstruction system, the fringe image is projected by the projector. When the fringe pattern is projected on the railway fastener, the deformed pattern, which carries the height information for the fastener, will be captured by the camera. Then, by means of triangulation, the point cloud map of the 3D topography of the fastener can be obtained in the world coordinate system.

In this system, the projected fringe images include four 90${}^{\circ }$ phase-shift sinusoidal fringe patterns, four Gray code patterns, one fully bright pattern, and one fully dark pattern. The method uses the Gray code auxiliary phase-shifting technology to avoid the discrete characteristics that occur when only Gray code projection is used, in order to improve the resolution of the 3D reconstruction system [19,20]. In contrast to the traditional line laser scanning method [13], this method only needs a few images to obtain the 3D topography of the entire fastener, which makes it faster. Moreover, compared with the 2D image photography and recognition method, it is stereoscopic and can obtain more subtle differences for each fastener. It is not limited to obvious states such as missing fasteners or damaged spring bars [5,6,7].

For Step *N*, the light intensity of the projection of the phase-shifting pattern can be described as [21]:

(1) $$I_{n}\left(x,y\right)=A\left(x,y\right)+B\left(x,y\right)\cos\left[\phi \left(x,y\right)+\frac{2\pi (n-1)}{N}\right]$$

where n=1,2,…,N is the index of the phase-shifting pattern, A(x,y) denotes the background light intensity, and B(x,y) is the light intensity modulation. ϕ(x,y) is the truncated phase with height information, which can be calculated using the deformed fringes obtained by projecting these *N* frames onto the object:

(2) $$\phi \left(x,y\right)=\tan^{-1}\left[\frac{\sum_{n=1}^{N}I_{n}\left(x,y\right)\sin\left(\frac{2\pi n}{N}\right)}{\sum_{n=1}^{N}I_{n}\left(x,y\right)\cos\left(\frac{2\pi n}{N}\right)}\right]$$

Owing to the operation of the arctangent function, this phase is periodically distributed. Therefore, the auxiliary projection Gray code can be used to obtain the level *k* where the phase is located and thus to acquire the global phase Φ(x,y):

(3) Φ(x,y)=ϕ(x,y)+2πk(x,y)

In the process of Gray code decoding, it is necessary to determine whether the current fringe is in a dark fringe or a bright fringe. Here, the approaches of Moreno and Taubin [22] and Nayar et al. [23] are adopted, and the direct and global light components formed during the projection are considered to reduce the interference caused by diffuse reflection from objects in ambient light.

After the global phase map is obtained, the method described by Feng et al. [24] can be used to calibrate and perform 3D reconstruction for the camera-projector system. Without considering the distortion of the camera and projector, let the coordinates of a point *P* on the railway fastener in the world coordinate system $O_{w}-X_{w}Y_{w}Z_{w}$ be $P_{w}={\left(x_{w},y_{w},z_{w}\right)}^{T}$. Therefore, the transformation of point *P* from the world coordinate system to the pixel plane coordinate system $O_{c}-X_{c}Y_{c}$ of the camera can be described by the following equation:

(4) $$s_{c}\left[\begin{array}{c} x_{c}\\ y_{c}\\ 1 \end{array}\right]=K_{c}\left[R_{c}|t_{c}\right]\left[\begin{array}{c} x_{w}\\ y_{w}\\ z_{w}\\ 1 \end{array}\right]$$

where $s_{c}$ is a scalar factor; $x_{c}$ and $y_{c}$ are pixel coordinates in the horizontal and vertical directions, respectively; $R_{c}$ is the rotation matrix in the camera pixel coordinate system and the projector pixel coordinate system; $t_{c}$ is the translation vector; and $K_{c}$ is the internal parameter matrix, describing the transformation of the projection from the camera coordinate system to the pixel plane coordinate system $O_{c}-X_{c}Y_{c}$. As the external parameter matrix, $\left[R_{c}|t_{c}\right]$ accomplishes the transformation from the world coordinate system to the camera coordinate system.

Similarly, the projector can be used as a camera with a reverse optical path, and thus an analogous model can be constructed in the same way:

(5) $$s_{p}\left[\begin{array}{c} x_{p}\\ y_{p}\\ 1 \end{array}\right]=K_{p}\left[R_{p}|t_{p}\right]\left[\begin{array}{c} x_{w}\\ y_{w}\\ z_{w}\\ 1 \end{array}\right]$$

Let $A_{c}=K_{c}\left[R_{c}|t_{c}\right]$, $A_{p}=K_{p}\left[R_{p}|t_{p}\right]$. Before the 3D reconstruction, internal and external parameters can be obtained through the checkerboard calibration method [24] to obtain the specific values of these matrices. Let $A_{c}^{ij}$ denote the element of the $i_{th}$ row and $j_{th}$ column of $A_{c}$, and let $A_{p}^{ij}$ denote the element of the $i_{th}$ row and $j_{th}$ column of $A_{p}$. By combining Equations (4) and (5), the 3D world coordinate point $P_{w}$ can be calculated as:

(6) $$\left[\begin{array}{c} x_{w}\\ y_{w}\\ z_{w} \end{array}\right]={\left[\begin{array}{ccc} A_{c}^{11}-x_{c}A_{c}^{31} & A_{c}^{12}-x_{c}A_{c}^{32} & A_{c}^{13}-x_{c}A_{c}^{33}\\ A_{c}^{21}-y_{c}A_{c}^{31} & A_{c}^{22}-y_{c}A_{c}^{32} & A_{c}^{23}-y_{c}A_{c}^{33}\\ A_{p}^{11}-x_{p}A_{p}^{31} & A_{p}^{12}-x_{p}A_{p}^{32} & A_{p}^{13}-x_{p}A_{p}^{33} \end{array}\right]}^{-1}\left[\begin{array}{c} x_{c}A_{c}^{34}-A_{c}^{14}\\ y_{c}A_{c}^{34}-A_{c}^{24}\\ x_{p}A_{p}^{34}-A_{p}^{14} \end{array}\right]$$

With this equation, the global phase $\Phi (x_{c},y_{c})$ of the projection corresponding to a pixel point ${\left(x_{c},y_{c}\right)}^{T}$ acquired by the camera can be calculated through the projected fringe. Then, $x_{p}$ can be obtained by the following equation, in which *W* is the pixel width of the fringe projected from the projector:

(7) $$x_{p}=\frac{\Phi (x_{c},y_{c})}{2\pi }W$$

The above steps can be summarized as follows. First, the system is used to generate Gray code and sinusoidal phase-shifting fringes. The projection is performed and captured by the camera, and from these images, the Gray code is decoded. Meanwhile, the truncated phase $\phi (x_{c},y_{c})$ can be obtained by Equation (2), and the global phase $\Phi (x_{c},y_{c})$ can be obtained by combining the Gray code and phase-shifting fringes (Equation (3)). Finally, the dense 3D point cloud map of the railway fastener is obtained using Equations (6) and (7) for subsequent point cloud processing and estimation of the clamping force.

### 3.2. Clamping Force Estimation Algorithm

#### 3.2.1. Point Cloud Denoising

After the point cloud map is obtained by calculation, it is necessary to preprocess the data to handle the noise points generated by hardware or software during the generation of the point cloud. There are many methods to remove point cloud noise, including moving least squares (MLS) methods, non-local methods, deep learning methods, and sparsity-based methods [25,26]. In selecting the processing method, considerations should include not only the denoising effects of the method but also two objectives: (1) that the amount of calculation during processing should be kept low to facilitate batch detection operations along the rail, and (2) that as the deformation of the railway fastener spring bar caused by different clamping forces is quite small, the coordinates of each point should remain unchanged to the extent possible during denoising, and outliers that could cause deviations in the subsequent clamping force estimation should be removed. With these objectives in mind, the statistical outlier removal algorithm [27] was adopted for the method developed in this study. It calculates the average distance $\mu_{i}$ from each point $P_{i}$ to the *k* adjacent points by performing neighborhood statistics on each point. It then calculates the mean $\mu_{all}$ and standard deviation $\sigma_{all}$ of the average distance of all points. If the mean distance $\mu_{i}$ of a point $P_{i}$ is outside the range $\mu_{all}\pm \alpha \sigma_{all}$, the point is considered an outlier and is removed. Here, *k* and α are parameters that can be set by the user. After selection and observation of different parameters, the values chosen in this study were k=100,α=0.5. In this way, the spatial noise points caused by external interference (such as illumination and acquisition) are filtered out, and the point cloud is complete.

#### 3.2.2. Point Cloud Registration and Specific Region Selection

After denoising is applied, point cloud registration is used to align the position and attitude of the point cloud map of the railway fastener with the railway fastener itself. This operation is done to facilitate the subsequent horizontal comparisons with the point cloud under various clamping states. Considering the need for batch detection, it should be noted that because there are differences between different railway fasteners, the point cloud registration algorithm needs to be highly robust. If the common ICP algorithm is used directly, it can easily become trapped in local convergence, leading to matching failure. Therefore, the point cloud registration algorithm is concerned only with the consistency of the shape of the spring bars for the various kinds of railway fasteners. In the algorithm, a typical spring bar part of the fastener is first selected as the reference point cloud. Then, coarse registration of the fasteners is conducted according to the FPFH features of the fasteners, followed by fine registration using the ICP algorithm. Finally, the irrelevant parts of the fastener point cloud are removed for the calculation of the clamping force; only the spring bar part is retained. The ROI of the fastener is selected by rectangular region selection. For WJ-8 fasteners, the length, width, and height of the spring bar parts are 140.7, 86.1, and 20.4 mm, respectively. In the following two sections, the coarse and fine registration algorithms are described separately.

**Figure 3:**
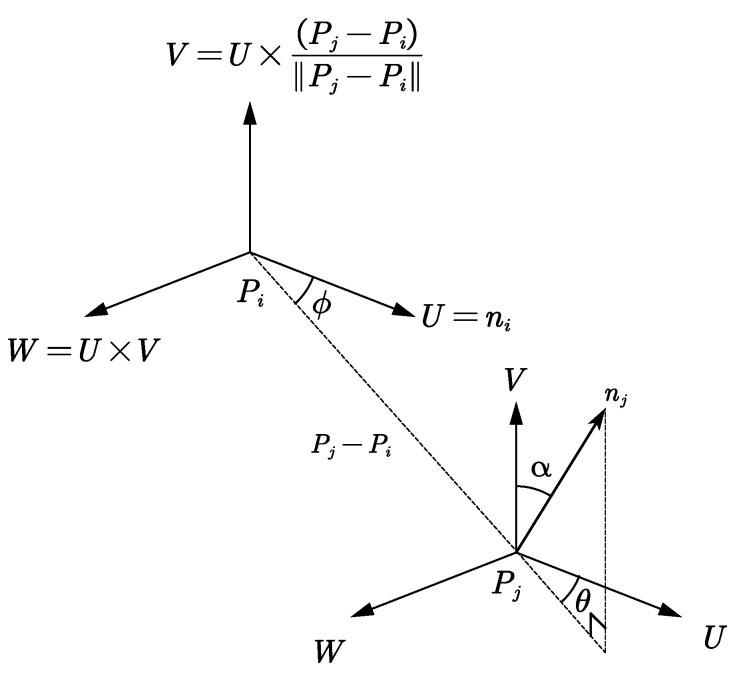
Definitions of point pair eigenvalues of points $P_{i}$ and $P_{j}$.

1. Coarse Registration Using FPFH
  In the FPFH algorithm, the estimated normals of point clouds are used to calculate the local features of the point clouds. It is an improved version of the point feature histograms (PFH) algorithm [28]. FPFH feature descriptors are robust under different sampling densities. In the method described in this study, the FPFH features of the reference point cloud and those of the point cloud to be matched are calculated, then the method proposed by Zhou et al. [28] is used to find the matching features between the two. After this, the coarse registration of the point cloud is conducted. For the calculation of FPFH features, three feature values of a pair of points in the point cloud need to be calculated. Suppose that points $P_{i}$ and $P_{j}$ have normals $n_{i}$ and $n_{j}$ in their neighborhoods. Then, a local coordinate system $P_{i}-UVW$ can be established on one of the points $P_{i}$, as shown in Figure 3. Based on the positions of the normals and the two points, three angles can be obtained, and then the eigenvalues (α,ϕ,θ) used by FPFH can be obtained as:
  (8) $$\alpha =V\cdot n_{j},\;\phi =U\cdot \frac{P_{j}-P_{i}}{∥P_{j}-P_{i}∥},\;\theta =\tan^{-1}\left(\;W\cdot n_{j},U\cdot n_{j}\right)$$
  The feature descriptor is calculated as follows.
  Step 1: Solve the surface normal $n_{i}$ for each point $P_{i}$ in the point cloud.
  Step 2: Calculate the point pair feature (α,ϕ,θ) for each point $P_{i}$ and the *k* adjacent points. Place the three values in a histogram by normalizing each eigenvalue and then apportioning them into *b* intervals of the same size, thereby obtaining a histogram with $b^{3}$ intervals formed by the combinations of the three sets of intervals. The histogram generated by these *k* point pair features can be used as a simplified point feature histogram (SPFH) for point $P_{i}$.
  Step 3: Generate the SPFH for each of the *k* points adjacent to $P_{i}$.
  Step 4: Generate the final FPFH descriptor of $P_{i}$ based on SPFH features with different weights:
  (9) $$FPFH\left(P_{i}\right)=SPFH\left(P_{i}\right)+\frac{1}{k}\sum_{j=1}^{k}\left(\frac{1}{w_{j}}SPFH\left(P_{j}\right)\right)$$
  where the weight $w_{j}$ is the distance between point $P_{i}$ and the adjacent points.
2. Fine Registration Using ICP Algorithm: After FPFH
  After FPFH coarse registration has been performed, the two point clouds will or should roughly coincide; then, the iterative closest point (ICP) algorithm is applied for fine registration. This algorithm solves a rigid body transformation R,t to minimize the following objective functions:
  (10) $$\min_{R,t}J=\frac{1}{2}\sum_{i=1}^{n}{∥P_{i}-RQ_{i}-t∥}^{2}$$
  where $P_{i}$ and $Q_{i}$ are the set of matching points in the point cloud to be registered and the reference point cloud. The singular value decomposition (SVD) method or the nonlinear optimization method can be used to minimize this function. Details of the algorithm are given in the paper by Besl and McKay [29].

#### 3.2.3. Comparisons with Multiple Reference Point Clouds

After registration of the point cloud from which the clamping force is detected and the spring bar region selected, a horizontal comparison can be performed between this point cloud and the point cloud under different clamping states. The reason that horizontal comparison was adopted is that an assumption is made that under the same or similar clamping forces, the shapes of different railway fasteners of the same model are fairly similar. Therefore, this distances resulting from the point cloud comparison can be used to measure the similarities between the point cloud of the fastener to be detected and the point cloud under different clamping force conditions. Then, the clamping force for the point cloud of the fastener can be detected. In this study, the absolute distance for each point in the point cloud comparison was obtained by horizontal comparison. The distribution of the absolute distances can be shown in the form of a histogram, and its distribution law can be used to establish a mathematical model for the estimated clamping force.

There are many methods available for estimating the distance between multiple point clouds. Methods that calculate the distance between points include Hausdorff distance estimation, multiscale model-to-model cloud comparison (M3C2) distance estimation, Wasserstein distance estimation, and Chamfer distance estimation [30,31]. There are also methods that estimate distances by fitting surface models such as planes, by Delaunay triangulation, or using quadratic surfaces in local regions [32]. Jafari et al. [30] used a method combining direct point-by-point distance measurement and statistical sampling to extract structural deformation information and quantified the strain and stress of mechanical deformation by comparing the results. They compared the advantages and disadvantages of Hausdorff distance estimation and M3C2 distance estimation and found that the M3C2 method produced more accurate and robust results. Urbach et al. [31] studied a new deep learning method for measuring the distance between point clouds, called the deep point cloud distance (DPDist), which is a significant improvement over the Chamfer distance estimation method for comparing similar objects. Ahmad Fuad et al. [32] applied point cloud comparison to landslide monitoring. They comprehensively evaluated Hausdorff distance estimation and the surface model-fitting method for estimating distance in local areas. They found that the plane-fitting method of estimating point cloud distance showed the lowest standard deviation when used in monitoring a landslide area. Because in an actual batch inspection work environment it is necessary to make comparisons between multiple fasteners within a short period of time, the method used in this study adopts the method using a local-area plane-fitting model. This is simple and accurate in statistical terms. Compared with quadric surface fitting, Delaunay triangulation, and other methods, it requires less computation. This method operates as follows.

Step 1: Define a neighborhood with radius $r_{c}$ for point $P_{i}$ in the point cloud to be compared and obtain the point set $Q_{c}=\left\{q_{1},q_{2},\ldots ,q_{n}\right\}$ of the reference point cloud in that neighborhood.

Step 2: Use the points in $Q_{c}$ for plane fitting. The fitting can be performed by using principal component analysis (PCA) or the random sample consensus method (RANSAC). In this study, PCA was used for the overall efficiency of the algorithm. Create the covariance matrix $G_{c}$ from $Q_{c}$:

(11) $$G_{c}=\frac{1}{n}\sum_{i=1}^{n}\left(q_{i}-\bar{q}\right){\left(q_{i}-\bar{q}\right)}^{T},\;\bar{q}=\frac{1}{n}\sum_{i=1}^{n}q_{i}$$

where $\bar{q}$ is the centroid of the local point cloud $Q_{c}$. $G_{c}$ is a 3×3 real symmetric matrix. The Jacobi method is used to analyze the eigenvalues and eigenvectors, thus obtaining three eigenvalues and eigenvectors of $G_{c}$. If $v_{min}$ is the eigenvector corresponding to the smallest of the three eigenvalues, then $v_{min}$ is the normal vector of the fitted plane

Let the equation of the fitting plane be Ax+By+Cz=D, the normal vector obtained be $v_{min}={\left(v_{x},v_{y},v_{z}\right)}^{T}$, and the centroid be

$$\bar{q}={\left({\bar{q}}_{x},{\bar{q}}_{y},{\bar{q}}_{z}\right)}^{T}.$$

Then

(12) $$A=\frac{v_{x}}{∥v_{\min}∥},\;B=\frac{v_{y}}{∥v_{\min}∥},\;C=\frac{v_{z}}{∥v_{\min}∥},\;D=A{\bar{q}}_{x}+B{\bar{q}}_{y}+C{\bar{q}}_{z}$$

Step 3: Obtain the distance $d_{i}$ between the point $P_{i}={\left(x_{i},y_{i},z_{i}\right)}^{T}$ in the point cloud to be compared to the fitted plane by using the following equation:

(13) $$d_{i}=Ax_{i}+By_{i}+Cz_{i}-D.$$

Step 4: Repeat Steps 1–3 to obtain the comparison distances for all points in the point cloud to be compared.

#### 3.2.4. Estimation of Point Cloud Distance Distribution

The number of distance values obtained by the comparisons between multiple fasteners is enormous; therefore, it is necessary to compress the data. As the comparison distance values display a regular distribution in the histogram, the kernel density estimation method is used to estimate the probability distribution values in a given interval. Then, a regression analysis is carried out with the probability distribution values. The kernel density estimation method was adopted because, being a non-parametric estimation method, it is well suited for fitting the distribution in the histogram of the comparison distances for fasteners under various conditions. In contrast to methods that estimate parameters by assuming a particular type of probability distribution, this method does not require any prior knowledge, and it has better adaptability for working with actual distributions [33,34].

The kernel density estimator uses the following formula to estimate the probability density function of the comparison distance:

(14) $${\hat{f}}_{h}\left(d\right)=\frac{1}{nh}\sum_{i=1}^{n}K\left(\frac{d-d_{i}}{h}\right)$$

where $d_{1},d_{2},\ldots ,d_{n}$ are the comparison distance values for the point cloud of a given fastener as calculated according to the local-area plane-fitting model. This is described in Section 3.2.3, with the number of points in the fastener point cloud that participate in the comparison being *n*, the kernel function K(·), and the bandwidth of the kernel function window *h*.

The existence of the kernel function requires that the following conditions be satisfied:

(15) K(u)≥0,∫K(u)du=1.

For this study, the kernel function selected was the Gaussian kernel function, which is defined as:

(16) $$K\left(u\right)=\frac{1}{\sqrt{2\pi }}e^{-\frac{1}{2}u^{2}}.$$

According to kernel density estimation theory [33], the bandwidth *h* of the window will affect the accuracy of the kernel density estimation. If the value of *h* is too large, it will lead to an ${\hat{f}}_{h}\left(d\right)$ curve that is too smooth and a large estimation error. If the value of h is too small, it will lead to large fluctuations in the ${\hat{f}}_{h}\left(d\right)$ curve. To avoid these problems, various methods have been proposed to adaptively select windows of different sizes according to the characteristics of the sample. This study adopted the rule-of-thumb formula commonly used for the selection of *h* [35]:

(17) $$h={\left(\frac{4{\hat{\sigma }}^{5}}{3n}\right)}^{\frac{1}{5}}\approx 1.06\hat{\sigma }n^{-1/5}$$

where $\hat{\sigma }$ is the standard deviation of the comparison distance values for the fastener point cloud.

#### 3.2.5. Regression Analysis

Using the method described in the previous section, the probability density function of the comparison distance between the fastener to be tested and the reference fastener under different clamping states is estimated. In order to perform the regression and estimation, additional measures are required to measure the clamping force of the fastener. The relationship between the tightening torque and the bolt clamping force is approximately linear [36,37] and can be described by the following formula. The formula adopted for this study was to use a torque wrench with a torque sensor to measure the tightening torque of the screw and then calculate the clamping force of the fastener indirectly.

(18) $$F=\frac{2T}{\frac{d_{2}}{\cos\alpha }\mu_{s}+\frac{P_{d}}{\pi }+d_{w}\mu_{w}}$$

The tightening torque of a bolt, denoted as *T*, is directly related to the clamping force, *F*, that the bolt produces. The middle diameter of the bolt is denoted as $d_{2}$, while the flank angle of the thread is represented by α. The friction coefficient of the thread is denoted as $\mu_{s}$, and the pitch of the thread is represented by $P_{d}$. Additionally, the equivalent friction diameter of the nut support surface is denoted as $d_{w}$, and the friction coefficient between the nut and the support surface is represented by $\mu_{w}$.

Suppose the clamping force values $F_{1},F_{2},\ldots ,F_{m}$ of *m* different fasteners are obtained using the torque wrench, and the probability density function family ${\hat{f}}_{h}{\left(d\right)}_{j}^{1},\ldots ,{\hat{f}}_{h}{\left(d\right)}_{j}^{k}$ of the comparison distance between a single force value $F_{j}$ and the reference fastener in *k* different states is calculated. Then, a ridge regression model can be established to predict the clamping force of the fastener to be tested. First, it is necessary to discretize the probability density function of the kernel density estimation, which can be evaluated at equidistant points starting from 0. That is, the following matrix can be calculated as the input variable for a single fastener to be tested:

(19) $$X_{j}=\left[\begin{array}{cccc} {\hat{f}}_{h}{\left(0\right)}_{j}^{1} & {\hat{f}}_{h}{\left(c\right)}_{j}^{1} & \cdots & {\left.{\hat{f}}_{h}\left[\left(N-1\right)c\right)\right]}_{j}^{1}\\ {\hat{f}}_{h}{\left(0\right)}_{j}^{2} & {\hat{f}}_{h}{\left(c\right)}_{j}^{2} & \cdots & {\left.{\hat{f}}_{h}\left[\left(N-1\right)c\right)\right]}_{j}^{2}\\ \vdots & \vdots & \ddots & \vdots \\ {\hat{f}}_{h}{\left(0\right)}_{j}^{k} & {\hat{f}}_{h}{\left(c\right)}_{j}^{k} & \cdots & {\left.{\hat{f}}_{h}\left[\left(N-1\right)c\right)\right]}_{j}^{k} \end{array}\right]$$

where 0,c,2c,…,(N−1)c are a series of equidistant points, and *c* and *N* are constants. Then, $X_{j}$ is transformed into a row vector: $X_{j(k\times N)}\to X_{j(l\times kN)}$. At this point, the observation matrix $X={\left[X_{1},X_{2},\ldots ,X_{m}\right]}^{T}$ and the response matrix $Y={\left[F_{1},F_{2},\ldots ,F_{m}\right]}^{T}$ can be constructed from the *m* observation samples $X_{j(l\times kN)}$, and then the regression model can be constructed using the two matrices.

Based on the actual calculation of characteristic roots of the matrix $X^{T}X$ (described in Section 4.4), it can be seen that the observation matrix X is ill-formed and that there is severe multicollinearity among the variables. This leads to too high a variance in the regression parameter estimates and poor estimation stability. In this situation, ridge regression, principal component regression, or partial least squares regression can be used to eliminate the collinearity. In the actual prediction of the clamping force, ridge regression was applied as the regression and prediction method because in practice it achieves a small root mean square error (RMSE) in most cases. The main concept of the ridge regression algorithm is that, in the presence of multicollinearity in the independent variables, $\left|X^{T}X\right|\approx 0$, and if a matrix $k_{rid}I$ (where ridge parameter $k_{rid}>0$) is introduced at this time, the degree to which the matrix $X^{T}X+k_{rid}I$ approaches singularity will be much smaller. The regression parameters at this time are:

(20) $$b_{\left(k_{\mathrm{rid}}\right)}={\left(X^{T}X+k_{\mathrm{rid}}I\right)}^{-1}X^{T}Y.$$

Then, if the observed variable for a scanned fastener point cloud is obtained as $X_{ob}$ using this algorithm, the final estimated clamping force will be:

(21) $$\hat{y}=b_{\left(k_{\mathrm{rid}}\right)}X_{\mathrm{ob}}.$$

The choice of value for the ridge parameter $k_{rid}$ is not unique. The ridge trace method and the variance expansion factor method can be used to select an appropriate value for $k_{rid}$, enabling the algorithm to obtain the optimal estimation model [38,39].

In practice, because the clamping force is random, the shape of the fasteners is random as well. Thus, we must consider the relationship between different forces and their shapes in random situations. In the study by Zeng et al. [40], they found that the growth rate of the longitudinal resistance of the fastener was independent of the vertical loading within the normal working range of the fastener. The relationship between fastener displacement and longitudinal load force is also linear for the same clamping torque. As a result, it can be assumed that the shapes of the fasteners are similar for the same cases of tie pressure, which will not have a large impact on the prediction of the algorithm. In order to verify the reliability of the algorithm, a random clamping torque will be applied to the fasteners in Section 4.5 for algorithm evaluation.

## 4. Testing, Results, and Analysis

In the experiments described in this section, multiple WJ-8 spring bar fasteners were tested to build a test model and assess the performance of the algorithms. First, the measurement accuracy of the 3D reconstruction system is evaluated. Next, the performance of the point cloud denoising and point cloud registration is reported. Then, point clouds are compared between multiple fasteners with the same fastener in different tightening states, illustrating the feasibility of estimating the railway fastener clamping force. Finally, the results obtained using different regression methods are compared.

### 4.1. Evaluation of the 3D Reconstruction System

Considering the fact that the elastic deformation of fasteners in different states of tightness is extremely small, it is necessary to ensure that the measurements by the 3D reconstruction system have an overall accuracy of a millimeter or better. A Canon EOS 800D camera with an image resolution of 1280 × 720, along with an Epson EF-10 projector with a fringe resolution of 1920 × 1080, were used to support the measurement accuracy desired for the 3D reconstruction system described in this paper. The fringe projection and acquisition software were written in C#, and the 3D reconstruction algorithm was run using MATLAB R2022a.

**Figure 4:**
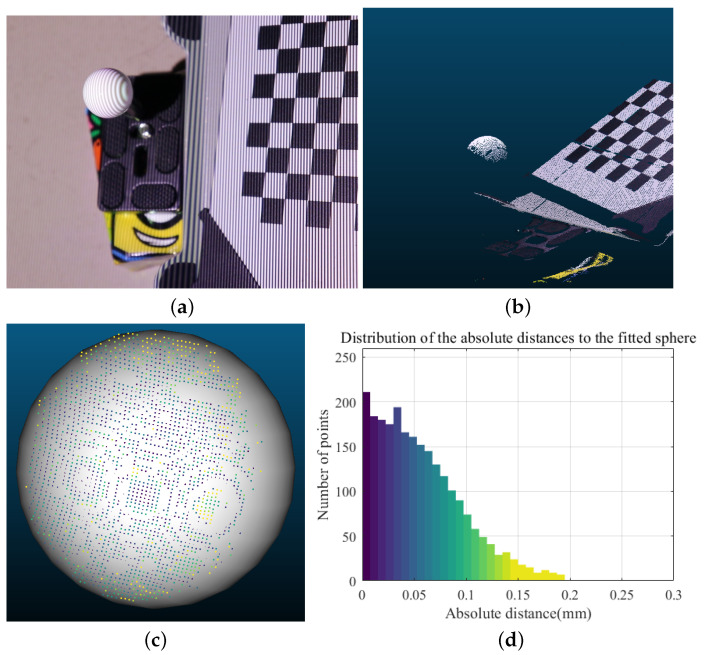
Evaluation of accuracy using a standard sphere. (**a**) Digital grating projection. (**b**) Point cloud obtained by system scanning. (**c**) Result of point cloud fitting to the standard sphere. (**d**) Histogram of the distances from the standard sphere to the fitted sphere.

A standard sphere with a radius of 10 mm was scanned to assess the measurement accuracy of the 3D measurement system. Figure 4a shows the content captured by the camera during the fringe projection. The white point cloud on the left is the standard sphere scanned, and on the right is the checkerboard calibration plate used in the system, each of whose squares has an edge length of 10 mm. The result after 3D scanning and denoising is shown in Figure 4b. The white point cloud was manually segmented out using CloudCompare software v2.12, and then a spherical fit was performed on it using the RANSAC algorithm, with the results shown in Figure 4c. The coloration shows the distribution of the absolute distances from the point cloud to the fitted sphere. The histogram showing the distribution of absolute distances is displayed in Figure 4d. From the histogram, the absolute distance from most points to the sphere is mostly within 0–0.15 mm. Table 1 shows the accuracy of test results run 5 times based on the standard sphere. This table lists the number of scanning points of the standard sphere, the radius of the fitting sphere, the maximum error value, and the root mean square error (RMSE) of the fitting. The result from this table satisfies the accuracy requirements of the measurement and testing system. These results provide a solid grounding for estimating the railway fastener clamping force.

### 4.2. Point Cloud Preprocessing Experiment

**Figure 5:**
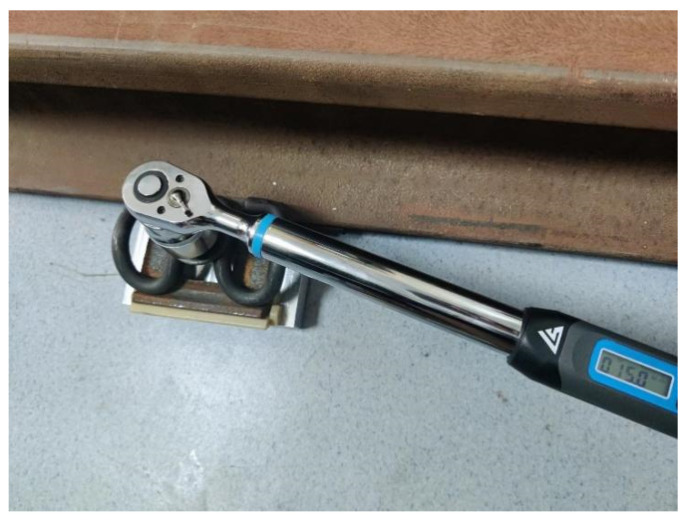
Torque wrench used to tighten a railway fastener.

In the method described in this paper, the system preprocesses the point cloud using point cloud denoising, coarse registration using FPFH features, fine registration using the ICP algorithm, and selection of the specific area of the point cloud. It does this to such a degree that the point cloud consistently maintains a uniform position and attitude before the comparison is performed. Only the spring bar of the fastener is processed. To obtain the measurement data for various clamping states, a torque wrench with a tightening torque sensor (Figure 5) was used to tighten 6 different fasteners 10 times each, with the tightening torque varying from 20 to 110 N·m in increments of approximately 10 N·m. After each tightening, the torque value displayed on the current sensor was recorded, and two 3D measurements from two different angles were taken using the 3D reconstruction system for evaluating system robustness. In addition, two 3D scans were performed on all the fasteners in a fully loosened state. Thus, a total of 132 3D scans were performed, providing sufficient data for subsequent point cloud data processing and regression. According to Chinese railway industry standard TB/T 3065-2002, the bolt model for WJ-8 railway fasteners is M24, and the tightening torque is required to be 100–140 N·m. The relevant parameters for the formula given by Equation (18) are as follows: d2 = 22.052 mm, α = 30${}^{\circ }$, $\mu_{s}\approx \mu_{w}\approx$ 0.14, $d_{w}$ = 36 mm, and $P_{d}$ = 3 mm. From these values, the clamping force corresponding to a tightening torque that ranges from 20 to 110 N·m was obtained as 4.18–23 kN.

**Figure 6:**
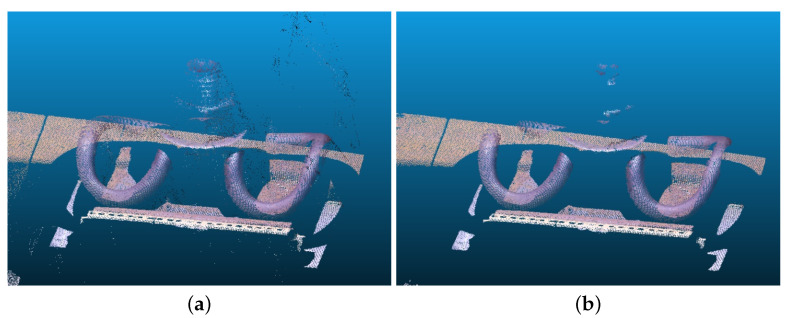
Denoising of the point cloud. (**a**) Original point cloud after the 3D reconstruction. (**b**) Result after point cloud denoising.

**Figure 7:**
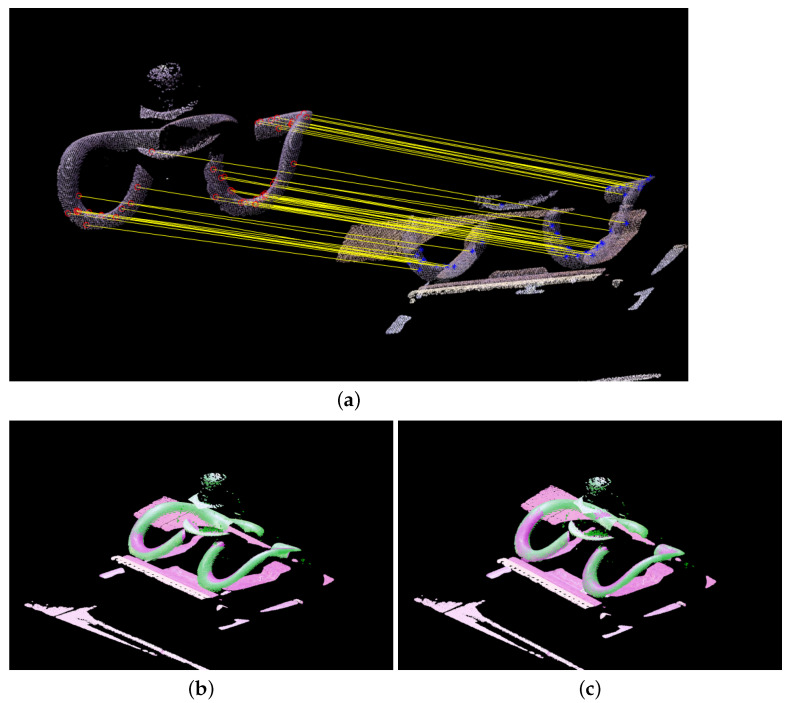
Registration of the point cloud. (**a**) Fast point feature histograms (FPFH) feature points matching the reference point cloud. (**b**) FPFH coarse registration result. (**c**) Iterative closest point (ICP) fine registration result.

The point cloud preprocessing is illustrated in Figure 6 and Figure 7. Figure 6a shows the point cloud of the railway fastener obtained from the 3D reconstruction system, and Figure 6b shows the point cloud after denoising, with 12,148 of the 121,917 points in the point cloud filtered out. Figure 7a shows the FPFH feature points obtained by the system from the matching between the point cloud to be measured and the reference point cloud. Figure 7b shows the result after FPFH coarse registration, and Figure 7c shows the result after ICP fine registration.

In order to further evaluate the registration algorithm in the system, this paper uses different registration methods to test separately, and the test results are shown in Table 2. The registration methods of Table 2 include the direct ICP method, fast global registration (FGR) [28], registration algorithm based on intrinsic shape signatures (ISS) [41], registration algorithm based on FPFH features, and FGR, ISS, and FPFH, respectively, combined with the ICP algorithm. Since the registration accuracy will significantly impact the following point cloud comparison algorithm, this paper regards the registration result with an RMSE greater than 1 mm as a registration failure. This paper counts the ratio of successful registration times in the 132 registrations. It can be seen from Table 2 that, for direct coarse registration methods, the FGR and ISS registration methods are faster than the ICP and FPFH methods. However, the registration accuracy is lower than theirs. The methods of coarse registration combined with fine registration have lower RMSE and higher registration success rates. The FPFH + ICP registration method has the highest registration success rate and the lowest RMSE. In order to make the prediction of the overall system that has higher accuracy, the system adopts the method of FPFH + ICP for registration.

For the accuracy registration algorithm ICP, it directly estimates the global attitude based on all the points. As a result, it runs for longer and is prone to local convergence, leading to poor agreement when more noise is present. For this reason, a combination of coarse registration and precision registration is typically used to perform the algorithm. In terms of three kinds of coarse registration algorithms, FGR, ISS, and FPFH, which all have similar basic ideas, are computed for the points first. Then, the point pairs with similar features to the two point clouds are calculated by certain methods, and finally the estimation of the pose matrix by the similar point pairs is performed. However, the FPFH algorithm retains more points than the ISS and FGR algorithms to then estimate the attitude matrix. For example, when the ISS extracts features from a point cloud, it excludes points that fall within a surface or lie on a line [41]. The FGR algorithm adopts dual verification and the distance difference verification method to exclude pairs of points when matching the feature points and prioritizes only the closest feature point when matching points are found [28]. The pre-exclusion and processing of points for both FGR and ISS reduces the computational load, leading to shorter run times than FPFH but also slightly reduced registration accuracy. The registration algorithm can also be chosen based on the actual accuracy and execution time requirements in the process of actually estimating the clamping force of the fastener.

### 4.3. Point Cloud Comparison Experiment and Analysis

**Figure 8:**
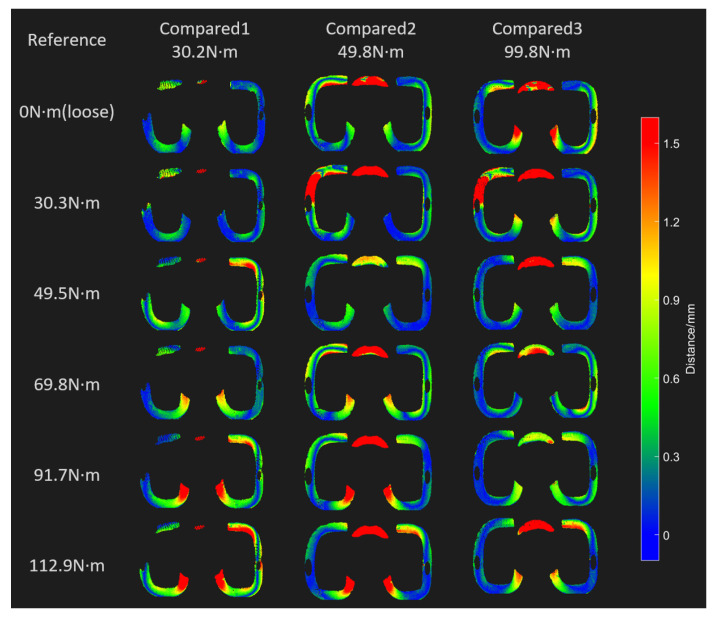
Horizontal comparison results for three railway fasteners.

**Figure 9:**
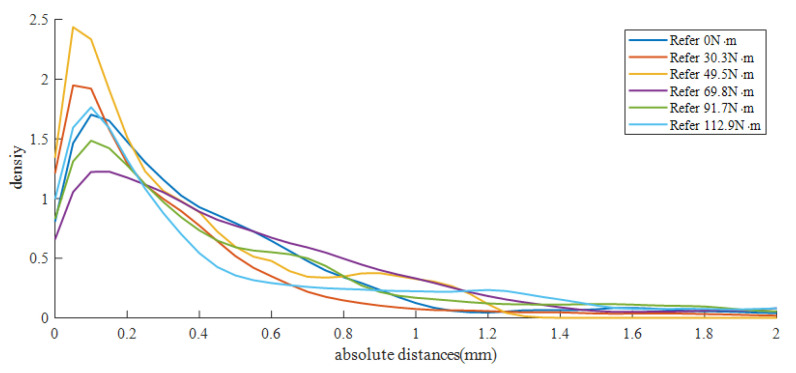
Kernel density estimations of the horizontal comparisons for a fastener tightened at 49.8 N·m.

Multiple fasteners were selected and compared with one fastener in various states of tightness to evaluate the feasibility of estimating clamping force by using horizontal comparisons. The partial results of the horizontal comparison of the three fasteners with tightening torques of 30.2, 49.8, and 99.8 N·m are shown in Figure 8, with different colors representing different comparison distance values. The closer the tightening torque value for the comparison fastener is to that of the reference fastener, the smaller the comparison distance value overall (i.e., the color becomes bluer). This finding is also in accordance with an intuitive understanding. This phenomenon is particularly obvious in the middle part of the fastener spring bar. For example, for the spring bar of the fastener tightened at 99.8 N·m, its middle part has a distance value distributed in the range of approximately 0.6–1.0 mm when compared with that of the fastener tightened at 91.7 N·m. This is less than the other comparison results in that column. Figure 9 shows the kernel density estimation results from the horizontal comparison between the 49.8 N·m fastener and the reference fastener. The values selected here are for c=0.05 and N=41, namely, equidistant values from 0 to 2 mm (references from Section 3.2.5 and Equation (19)). In actual testing, this range of values covers most of the comparison distance values under normal conditions. It can be seen from the figure that when the 49.8 N·m fastener is compared with the 49.5 N·m fastener, a larger kernel density estimation is obtained at a smaller distance value, and its distribution is more concentrated.

### 4.4. Analysis of Regression Effects

According to the analysis in the previous section, it can be seen that for the fasteners with a tightening torque close to that of the reference, the distribution of the measured comparison distance values is closer to 0 mm. therefore, it is suitable to perform the regression analysis using the kernel density estimation. Before the regression analysis, 11 measurement results for one fastener were selected from the 132 measurement results as the reference point cloud for horizontal comparison. Then, the 110 point clouds for the other five fasteners were used as the point clouds to be compared. A program was written to perform the horizontal comparison of the 110 point clouds and the kernel density estimation. Then, according to Equation (19), equidistant values were taken from the kernel density estimation function to obtain the input parameters $X_{1},X_{2},\ldots ,X_{110}$ for the regression. When c=0.05 and N=41, there are 451 input variables, greater than the number of observations. This regression problem is therefore a high-dimensionality data prediction problem, and there may be multicollinearity among the input variables. Considering this possibility, the characteristic roots of the matrix $X^{T}X$ were calculated to estimate the number of variables with collinearity [42] (there are as many multicollinearity relations in the observation matrix X as there are characteristic roots close to zero). After the calculation, 389 characteristic roots smaller than 0.001 were obtained, indicating the need to take extra measures to eliminate the collinearity.

**Figure 10:**
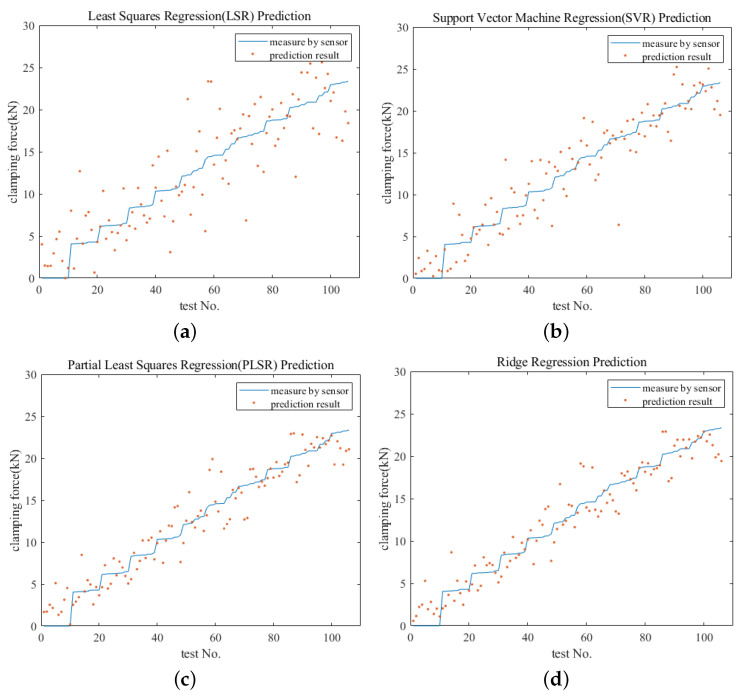
Comparison of predicted clamping force and actual measurement results. (**a**) Results predicted by LSR. (**b**) Results predicted by SVR. (**c**) Results predicted by PLSR. (**d**) Results predicted by ridge regression.

To make full use of the test samples, the leave-one-out cross-validation method was applied to the observation point cloud to establish a regression model and make the estimation. To eliminate the multicollinearity among variables, four regression models were applied: least squares regression (LSR) employing generalized inverse, support vector regression (SVR), partial least squares regression (PLSR), and ridge regression. Figure 10 shows comparisons between the predicted and the measured clamping force results for the test set constructed by the leave-one-out method under different tightness states. SVR, PLSR, and ridge regression all achieved relatively good regression results in the regression prediction, demonstrating the effectiveness of these three algorithms in eliminating collinearity. The results predicted by ridge regression came closer to the measured results than those of the other algorithms. Table 3 shows the maximum prediction error and root mean square error of the four prediction models. The algorithm in the ridge regression model achieves good measurement accuracy. Considering that the tightening torque was measured at intervals of approximately 10 N·m during data collection, the tightening torque RMSE of 9.274 N·m and the clamping force RMSE of 1.940 kN demonstrate that the prediction accuracy is sufficient to meet the requirements for actual application.

### 4.5. Evaluation of Algorithm

To further test and evaluate the reliability of the algorithm described in this paper, we tested the algorithm using three different tightening test methods similar to those described in Figure 5:

1. Tested by giving fasteners random stress. Because the elastic state of the rail fastener is often random, a random clamping torque can be applied and recorded, and then the algorithm can be predicted and evaluated. During the experiment, we applied a random loop pressure from 0 to 120 N·m to the fastener.
2. Testing the fastener in two states: full tightening and loosening. In a batch work environment, it is necessary to monitor whether the fasteners are in a tight or loose state condition, so this method can be used to evaluate whether the algorithm has a direct binary judgement. Tensile torque requirements for Type II fasteners range between 100 and 140 N·m, in line with the TB/T 3065-2002 standard of the Chinese rail industry mentioned above. In this paper, we use more than 100 N·m fasteners and fasteners in or near a loose state (tightening torque less than 30 N·m) for the algorithm prediction.
3. Test by gradually increasing the fastener stress. To compare the two methods, the fasteners are tightened in a step-by-step fashion. For each clamping torque, the torque was changed from 20 N·m to 110 N·m, increasing by approximately 10 N·m each time, with two 3D scans at different angles.

**Figure 11:**
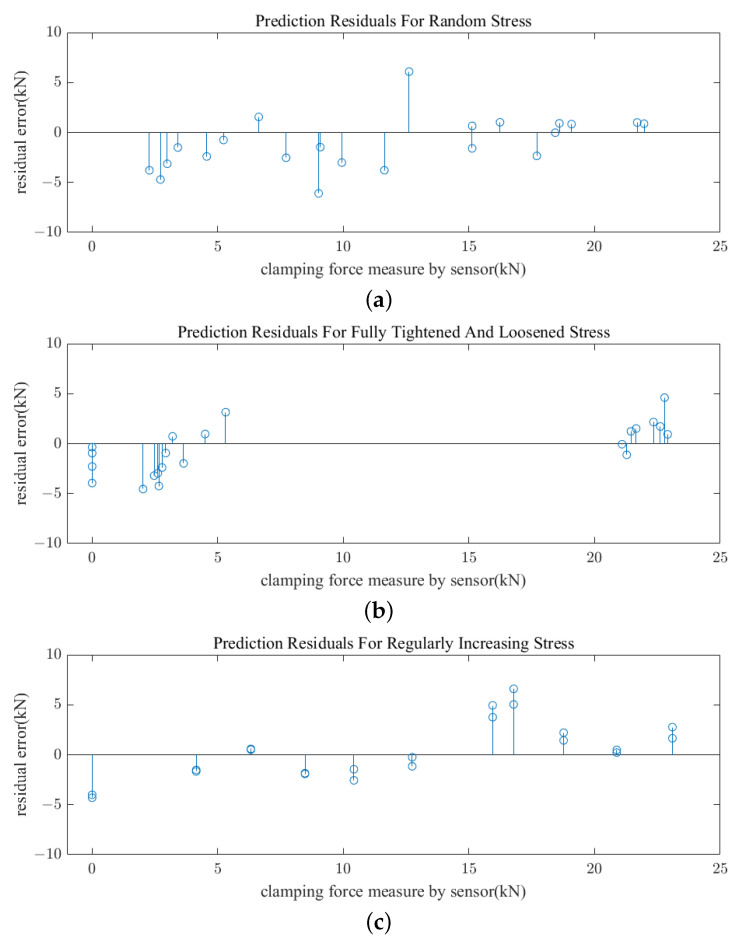
Prediction residuals with three testing approaches. (**a**) Random stress. (**b**) Fully tightened and loosened stress. (**c**) Regularly increase stress.

In this paper, the algorithm is evaluated by these three methods, each of which carries out 22 3D reconstruction and algorithm prediction. The residual difference between the actual measured and the predicted stress is shown in Figure 11, where the X-axis is the actual measured clamping force and the Y-axis is the predicted residual. Table 4 shows the computed RMSEs and average runtimes of the algorithms for predictions using the three methods. As can be seen, the prediction errors for the three test processes are close to one another, and the error of prediction is in a reasonable range of values. As for the second test method, it can be seen from the figure that the algorithm completely distinguishes the two states of fastener tightening and loosening conditions, indicating that there is no misjudgment in the prediction process.

## 5. Conclusions

The detection of buckle pressure in a railway system is a challenging task. In order to meet the demand of mass detection on a railway, a railway fastener pressure prediction system is designed in this paper. The system uses digital raster projection technology to measure the three-dimensional shape of the fastener. Then, the system predicts the actual buckle pressure of the railway fastener by a series of algorithms, such as point cloud denoising, point cloud rough registration based on FPFH characteristics of point cloud, ICP fine registration, point cloud specific region selection, kernel density estimation, and ridge regression. The actual test shows that the RMSE of the system is 1.94 kN for predicting the clamping force, which can meet the requirement of the buckle pressure measurement under normal circumstances. In order to evaluate the reliability of the algorithm, three different tightening methods are tested in this paper. The results show that the predicted results are within a reasonable range. Compared with the vibration signal detection, plane vision detection, laser scanning detection, and other methods used in the past, this method is more efficient and can describe the tightening state of railway fasteners more precisely, rather than being limited to the more intuitive cases such as missing or damaged fasteners.

However, there are still limitations to this method, which will be solved in future work. First, when the system performs 3D scanning, the projective light is non-linear, which will affect the actual measurement effect. In the future, some active or passive nonlinear correction methods are needed to compensate for the projected light. Secondly, the establishment of transverse comparison and regression models in the system requires a large number of observed data under different tightening states. In the future, adopting a direct method that can extract the shape curve of the spring bar from the 3D topography of the fastener for predicting the clamping force should be considered.

Zhengji Fan, YingPing Hong, Yunfeng Wang, Yanan Niu, Huixin Zhang and Chengqun Chu

## Figures and Tables

**Table 1:** Accuracy test results based on a standard ball (radius 10 mm).

Scan No.	No. of Scan Points	Radius of Fitted Sphere (mm)	Maximum Error (mm)	RMSE ${}^{*}$ of Fit (mm)
1	2501	10.0863	0.3141	0.0566
2	2920	10.0175	0.4860	0.0824
3	3019	10.0991	0.5953	0.0585
4	2975	10.0252	0.3144	0.0584
5	3055	10.0357	0.3152	0.0532

* RMSE, root mean square error.

**Table 2:** Test results of different registration methods and their combinations.

Registration Method	No. of Tests	No. of Registration Failures	Successful Registration Rate	Average Time Spent onRegistration (s)	Average Registration RMSE (mm)
ICP	132	7	94.70%	2.7121	0.4464
FGR	132	125	5.30%	0.5403	1.5622
ISS	132	65	49.24%	0.3558	1.3390
FPFH	132	2	98.48%	1.1528	0.5210
FGR + ICP	132	2	98.48%	1.7718	0.3587
ISS + ICP	132	1	99.24%	1.8444	0.3467
FPFH + ICP	132	0	100%	2.4011	0.3292

**Table 3:** Maximum prediction error and root mean square error of four prediction models.

Regression Method	Tightening Torque (N·m)		Clamping Force (kN)	
	Maximum Error	RMSE	Maximum Error	RMSE
LSR	72.504	19.505	15.169	4.080
SVR	49.664	11.923	10.390	2.494
PLSR	26.225	9.744	5.486	2.038
Ridge Regression	25.302	9.274	5.293	1.940

**Table 4:** Root mean square error and average prediction time of three test methods.

Test Method	Tightening Torque RMSE (N·m)	Clamping Force RMSE (kN)	Average Time Spent on Prediction (s)
Random Stress	13.530	2.831	24.53
Fully tightened and loosened stress	11.887	2.487	23.16
Regularly increase stress	13.383	2.800	24.29

## Data Availability

Data will be made available on request.
